# LKB1: Can We Target an Hidden Target? Focus on NSCLC

**DOI:** 10.3389/fonc.2022.889826

**Published:** 2022-05-11

**Authors:** Gloriana Ndembe, Ilenia Intini, Elisa Perin, Mirko Marabese, Elisa Caiola, Paolo Mendogni, Lorenzo Rosso, Massimo Broggini, Marika Colombo

**Affiliations:** ^1^ Laboratory of Molecular Pharmacology, Istituto di Ricerche Farmacologiche Mario Negri IRCCS, Milan, Italy; ^2^ Thoracic Surgery and Lung Transplantation Unit, Fondazione Istituto di Ricovero e Cura a Carattere Scientifico (IRCCS) Ca’ Granda Ospedale Maggiore Policlinico, Milan, Italy; ^3^ Department of Pathophysiology and Transplantation, University of Milan, Milan, Italy

**Keywords:** non-small cell lung cancer, STK11 (LKB1), target, personalized medicine, metabolism

## Abstract

*LKB1* (liver kinase B1) is a master regulator of several processes such as metabolism, proliferation, cell polarity and immunity. About one third of non-small cell lung cancers (NSCLCs) present *LKB1* alterations, which almost invariably lead to protein loss, resulting in the absence of a potential druggable target. In addition, LKB1-null tumors are very aggressive and resistant to chemotherapy, targeted therapies and immune checkpoint inhibitors (ICIs). In this review, we report and comment strategies that exploit peculiar co-vulnerabilities to effectively treat this subgroup of NSCLCs. LKB1 loss leads to an enhanced metabolic avidity, and treatments inducing metabolic stress were successful in inhibiting tumor growth in several preclinical models. Biguanides, by compromising mitochondria and reducing systemic glucose availability, and the glutaminase inhibitor telaglenastat (CB-839), inhibiting glutamate production and reducing carbon intermediates essential for TCA cycle progression, have provided the most interesting results and entered different clinical trials enrolling also LKB1-null NSCLC patients. Nutrient deprivation has been investigated as an alternative therapeutic intervention, giving rise to interesting results exploitable to design specific dietetic regimens able to counteract cancer progression. Other strategies aimed at targeting LKB1-null NSCLCs exploit its pivotal role in modulating cell proliferation and cell invasion. Several inhibitors of LKB1 downstream proteins, such as mTOR, MEK, ERK and SRK/FAK, resulted specifically active on *LKB1*-mutated preclinical models and, being molecules already in clinical experimentation, could be soon proposed as a specific therapy for these patients. In particular, the rational use in combination of these inhibitors represents a very promising strategy to prevent the activation of collateral pathways and possibly avoid the potential emergence of resistance to these drugs. LKB1-null phenotype has been correlated to ICIs resistance but several studies have already proposed the mechanisms involved and potential interventions. Interestingly, emerging data highlighted that *LKB1* alterations represent positive determinants to the new KRAS specific inhibitors response in *KRAS* co-mutated NSCLCs. In conclusion, the absence of the target did not block the development of treatments able to hit *LKB1*-mutated NSCLCs acting on several fronts. This will give patients a concrete chance to finally benefit from an effective therapy.

## Introduction

*STK11* (serine-threonine kinase 11), also known as *LKB1* (liver kinase B1) is an oncosuppressor gene on the short arm of chromosome 19 (19p13.3), composed of 9 coding exons separated by 8 introns, encoding for the highly conserved serine threonine kinase LKB1 (1–4). *STK11* germline mutations were originally identified in the autosomal dominant Peutz-Jeghers Syndrome (PJS), a disease involving the formation of polyps in the gastrointestinal tract (5–7). Besides germline mutations, *LKB1* can be inactivated by somatic mutations that lead to a predisposition to sporadic cancers such as pancreatic, breast and gastrointestinal cancers, as well as melanoma and especially lung cancer. *STK11* is the third most frequently mutated gene NSCLC adenocarcinoma, accounting for 30% of cases (1, 8–14). In NSCLC, these mutations are associated with the development of particularly aggressive cancer phenotypes, with rapid cell proliferation and a high tendency to develop metastases (1, 8, 10, 15–20).

LKB1 is a 48 kDa protein of 433 amino acids and contains three domains: a C-terminal domain with regulatory function, a central kinase domain and an N-terminal nuclear localization signal (NLS) (2–4). To function LKB1 must be associated with STRAD and MO25, two proteins that promote its cytoplasmic localization and boost its catalytic activity (4, 21, 22). Through the activation of AMP-activated protein kinase (AMPK) and 13 other AMPK-related serine-threonine kinases, LKB1 regulates several key cellular pathways involved in the regulation of energy metabolism, cell proliferation, cell polarity, extracellular matrix adhesion, mitosis and DNA damage response (4, 23–30).

Under metabolic stress LKB1 activates AMPK, which inhibits anabolic pathways and stimulates catabolic ones to restore cell energy homeostasis. For example, the LKB1/AMPK pathway, through inhibition of the mammalian target of rapamycin (mTOR) complex, blocks cell proliferation and protein synthesis (24, 25). Moreover, experimental evidence indicates that LKB1/AMPK pathway also has a role in regulating epithelial tight junction assembly and disassembly and it is essential to ensure proper cell adhesion to the extracellular matrix (31, 32). LKB1 is also important in the regulation of cell polarity through the activation of some AMPK-related kinases such as MARK1, 2, 3, 4 and BRSK. In addition, a report showed that the *PAR-4* gene, required for establishing apical/basal polarity in C. elegans, encodes for a putative serine-threonine kinase, which has 42% amino acid identity in the kinase domain with LKB1. Therefore, this evidence corroborates the importance of LKB1 in inhibiting metastasis formation and tumor differentiation and invasion (21, 33–36).

In the nucleus LKB1 acts on mitosis. Inhibiting PLK1 (Polo-like kinase 1), it blocks centromere over duplication and by interacting with p53 it reduces survivin levels, avoiding mitosis disorders (37). LKB1 also has a key role in the DNA damage response: it directly promotes non-homologous end-joining (NHEJ) with consequent maintenance of genomic stability (38). In addition, after an increase in cellular oxidative stress caused by exposure to UV or ionizing radiation, ataxia telangiectasia mutated (ATM) activates LKB1 which, through AMPK-induced autophagy, restores cell homeostasis (38–42). In addition, STK11 is also active in modulation of the immune system. Its inactivation promotes the production of proinflammatory cytokines CXCL7, G-CSF and IL-6, which contribute to the accumulation of T cell suppressor neutrophils, with corresponding low CD4+/CD8+ T lymphocyte infiltration and low levels of PD-L1 (programmed cell death-1 ligand) (43–47).

Overall, *STK11* is essential in the regulation of all these pathways and it is clear that its mutations can have serious consequences on correct cellular function.

More than 400 *STK11* mutations have now been discovered, such as single nucleotide variation, indels, hypermethylation of the promoter and homozygous deletions (9, 48, 49), 70% of these mutations lead to the production of a truncated protein with complete loss of its catalytic function, while the remaining 30% are missense mutations (17, 48). However, as in almost all missense mutations, there is a complete loss of LKB1’s oncosuppressive function, since that these mutations directly affect its kinase domain or the domains that guarantee its cytoplasmic localization (9, 48, 49). These characteristics mean that *STK11* mutated tumors cannot be treated either with direct targeted therapy, because the protein is completely absent, or with immunotherapy alone because of the “cold” tumor immune microenvironment (43, 46, 50).

In the light of this evidence, the aim of this review is to highlight some strategies studied to target *LKB1*-mutated tumors exploiting the pathways altered by the absence of its protein.

We report first the strategies to target LKB1 exploiting the metabolic dysregulation, then second, strategies that specifically target proteins downstream of LKB1, and finally strategies involving the immune system.

## Targeting LKB1 in Metabolism

Aberrant cell metabolism is an important characteristic of cancer cells, and the rewiring of metabolic pathways is essential for the initiation, proliferation and progression of tumors (51). Actually, unlike normal one, cancer cells do not primarily rely on mitochondrial oxidative phosphorylation but instead use aerobic glycolysis to generate energy (Warburg effect) (52).

The link between LKB1 and cell metabolism, through the activation of AMPK, has acquired increasing evidence. AMPK is a central metabolic sensor which in ATP-low level conditions, induces the switch from anabolic to catabolic metabolism, by inhibiting the synthesis of fatty acids (FA), cholesterol, triglycerides, glycogen and proteins, besides increasing glycolysis and FA oxidation (53, 54). When activated, AMPK modulates the mTOR pathway, inhibiting mTOR complex I (mTORC1) through the activation of TSC2 (a negative mTOR regulator) and inhibition of the mTORC1 subunit RAPTOR (25).

**Figure 1:**
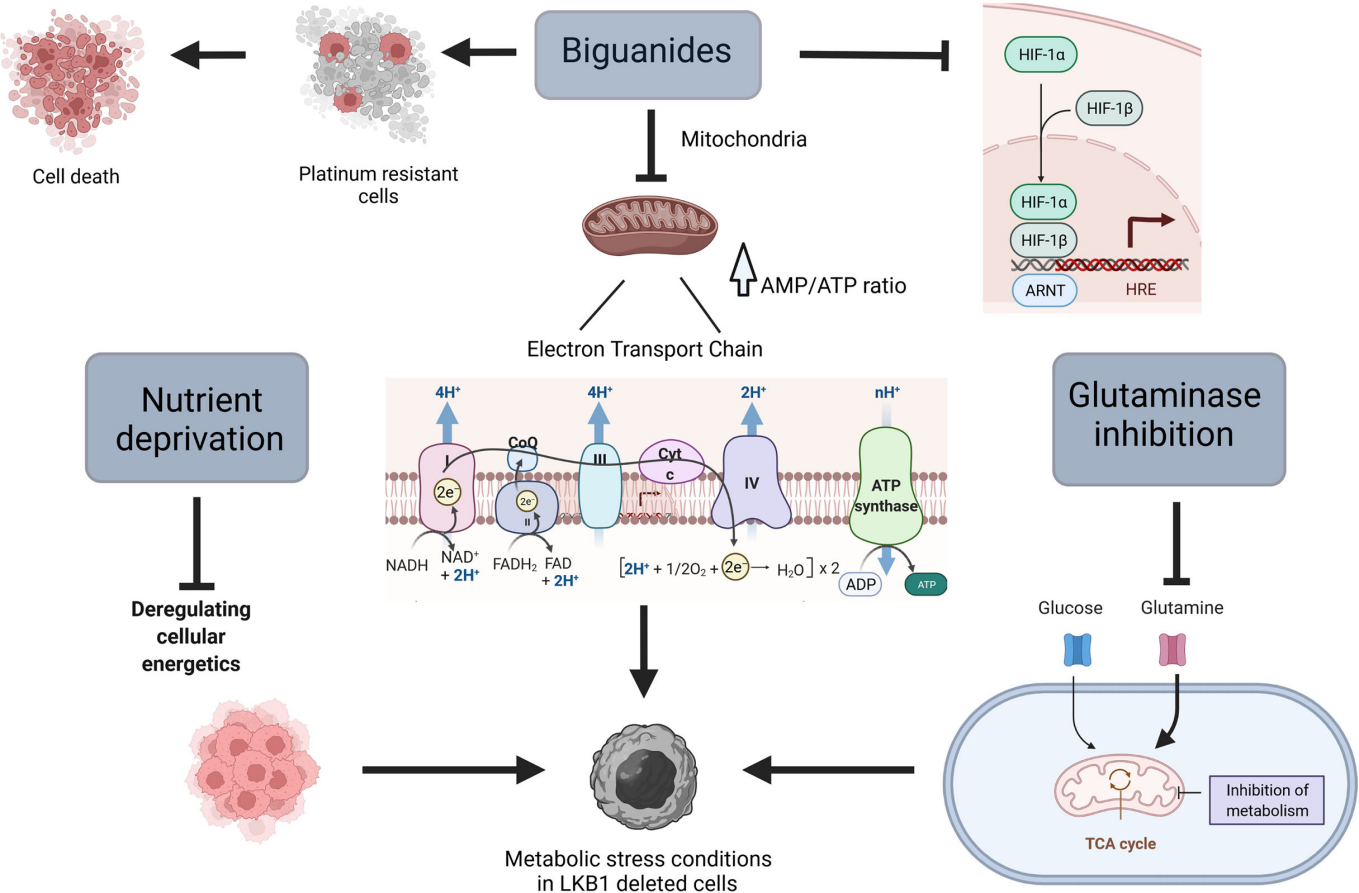
Scheme of different ways to target metabolism in *LKB1*-deleted cells. Created with Biorender.com.

Loss of LKB1 results in mitochondria and metabolic dysfunction that make cells unable to respond to metabolic stress (55–57), this is why tumors with this mutation might be considered a “niche”, selectively targetable with agents that lead to metabolic stress (**Figure 1**).

### Biguanides

Metformin is widely used as first line therapy in type 2 diabetes mellitus (58), but in the last few years it has attracted attention also for its anticancer potential. In 2005, a report showed the link between the reduction of cancer incidence and metformin treatment in diabetic patients, and subsequent studies have pointed out how the anticancer effect of this drug could be employed for both diabetic and non-diabetic patients (59).

At the cellular level, the primary target of metformin is the mitochondrion, in which it inhibits complex I of the mitochondrial electron transport chain, reducing cellular levels of adenosine triphosphate (ATP) and rising adenosine monophosphate (AMP). The increased of AMP/ATP ratio induces the activation of AMPK and the switch from anabolic to catabolic metabolism (60, 61). In a very recent study, Ma et al. underline an alternative mechanism of action played by metformin in an AMP-independent manner. In fact, they underline the ability of metformin to target PEN2, which in turn induces the activation of lysosomal AMPK (62). In view of these observations and given the key role of LKB1 in controlling metabolism homeostasis, different studies have indicated the possibility of using the biguanide metformin (and the related drug phenformin) in combination with first-line drugs, to induce metabolic stress *in vitro* and *in vivo* in cells lacking LKB1.

What emerges from NSCLC models mutated in *KRAS* and *STK11/LKB1* is greater sensitivity to phenformin due to both the constitutive activation of KRAS, which induces cell proliferation, and the absence of LKB1 which renders the cell unable to face metabolic stress (63). In pre-clinical experiments in NSCLC *KRAS*/*LKB1*, metformin synergizes with cisplatin, inducing selective activation of apoptosis and preventing platinum resistance (64). Interestingly, metformin induces apoptosis in NSCLC cells overexpressing miRNA-17, which has been identified as a negative regulator of LKB1 conferring a LKB1-less status on otherwise wt *LKB1* cells (65).

Different mechanisms of action explain the greater response induced by the biguanides in the absence of LKB1. The metabolic reprogramming in LKB1-deficient cells is shown to be dependent on hypoxia-inducible factor-1α (HIF-1α) (66). This transcription factor is regulated by oxygen availability and its protein expression is normally stabilized in hypoxic conditions. HIF-1α plays a pivotal role in the activation of different survival pathways in cancer cells (67). In line with these observations, tumor cells deleted in *LKB1* present an accumulation of HIF-1α also under normoxia (68). Metformin inhibits HIF-1α expression, reversing the metabolic advantage of LKB1-deficient cells (7, 66, 67).

MicroRNA-7 was identified as a tumor suppressor, important in the development of different solid tumors. Dong et al. underlined the upregulation of miRNA-7 mediated by metformin, inducing the suppression of proliferation and metastasis in a NSCLC cell line deleted in *LKB1* (69).

Luo et al. too showed that in NSCLC lacking LKB1 metformin enhances survivin degradation, through inhibition of the protein kinase A (PKA)/glycogen synthase kinase-3β (GSK-3β) axis, mediated by activation of AMPK (70). Survivin is one of the inhibitors of apoptosis (IAP) family (71), overexpressed in many tumors, and enables cells to avoid apoptosis (72). In addition, metformin reverses chemo-resistance in NSCLC LKB1-null cells, by stimulating nuclear factor erythroid 2-related factor 2 (Nrf2) degradation (73).

The antitumor activity of metformin might also be associated with its ability to lower circulating glucose and insulin levels, which are involved in tumor onset in some circumstances (74–76).

Several studies have examined the effect of the biguanides in combination with other agents that can induce metabolic stress, in different cancer models. Parker et al. reported that cells lacking LKB1 are more sensitive than LKB1-proficient cells to the combination of phenformin and a glutaminase inhibitor, being more dependent on reductive glutamine metabolism (55).

Clinical trials are testing the effect of metformin in various cancers. In particular, FAME trial aims to demonstrate whether metformin alone or in combination with a fasting-mimicking diet to the standard therapy, improves progression-free survival of patients with advanced, LKB1-inactive lung adenocarcinoma (77).

### Nutrient Deprivation

Nutrient deprivation is an emerging strategy to reduce the availability of metabolites, crucial for tumor cells, creating an environment that reduces cancer cells’ ability to adapt, thus increasing apoptosis and autophagy (78, 79). In addition, studies have shown that the combination of fasting chemotherapy, enhances the effect of the latter, reducing its side effects (79).

Different ways have been developed to lower the supply of nutrients to the tumor microenvironment, including caloric restriction, intermittent fasting and a newly designed fasting diet called ‘fasting-mimicking diet’ (FMD) (80). Caloric restriction is defined as a chronic 20-40% reduction of calorie intake (less than 200 kcal/day) without malnutrition, while intermittent fasting consists of total abstinence or minimal intake of food and drink for a period that typically ranges from 12 hours to 3 weeks, followed by the reintroduction of nourishment (81). To overcome side effects such as malnourishment, cachexia and the impairment of immune system, that may arise during fasting, FMD has been developed. As less restrictive than fasting, providing the essential nutrients for the body, while maintaining the beneficial effects of fasting (82).

Fasting directly regulates different pathways involved in cell metabolism such as SIRTs, NRF2, FOXO1, NFkB, HIF-1α as well as mTOR and AMPK, leading to the inhibition of cell cycle progression, glycogen synthesis, FA oxidation and angiogenesis, lowering the AMP : ATP and NAD : NADH ratios, glucose metabolism, cell growth and proliferation (83, 84). The reduction of nutrient concentration also changes the levels of circulating hormones and metabolites, resulting in a decrease of cell division in normal cells (85, 86). Cancer cells that continue to grow in a nutrient-deficient microenvironment become more sensitive to chemotherapy and other cancer therapies (87).

Caiola et al. observed that *KRAS/LKB1* NSCLC cells, not being able to compensate the increase of ATP demand under the mitochondrial oxidative phosphorylation system (OXPHOS) and glycolytic restriction, exploiting already at their maximum metabolic capacity, become susceptible to caloric restriction *in vitro* (78).

Clinical trials are currently investigating the effects of nutrient deprivation in various cancers. The ongoing FAME trial (77) is already assessing this directly in NSCLC and we have great expectations for the results.

### Glutaminase Inhibition

Glutamine metabolism is one of the major bioenergetic and biosynthesis supporter in tumors (88). Through its catabolism cancer cells can maintain pools of different carbon intermediates, that enter the TCA cycle (glutaminolysis) (89, 90). Glutaminolysis directly contributes to the regulation of redox homeostasis, besides than being involved in different aspects of tumor metastasis including epithelial-mesenchymal transition. In addition, there may be a link between glutamine metabolism and tumor microenvironment as well as tumor immunology (88, 91–94), though the exact mechanisms are still not known. In view of the importance of the glutamine metabolism in tumor growth and progression, in the recent years metabolic targeting therapies, alone or combined with chemo and radiotherapy, have been investigated in different cancer types (89, 95–98).

Glutaminolysis is regulated by different enzymes, glutaminase holding a key role. Inhibition of glutaminase reduces glutamine to glutamate conversion, resulting in less availability of carbon intermediates together with accumulation of intracellular ROS (96).

In NSCLC *STK11/LKB1* is often co-mutated with *KEAP1* (99), defining an additional subgroup. *KEAP1* is an oncogene that negatively regulates NRF2 by inducing its degradation through ubiquitination (100). Whereas tumors bearing the deletion in *STK11/LKB1* have high levels of reactive oxygen species (ROS) (39), it can be assumed that this condition leads to a positive selection pressure for *KEAP1* loss. Thus, *KEAP1* deletion provides a protection against ROS-mediated damage brokered by NRF2 (96). Galan-Cobo et al. reported an increase in the expression of genes involved in glutamine metabolism in lung adenocarcinoma cells with the mutations of *KRAS*, *STK11/LKB1* and *KEAP1*. In fact, *KRAS*/*LKB1*/*KEAP1* mutant tumors were found to be dependent on glutamine metabolism, unlike *KRAS* mutated tumors, and consequently they turned out to be sensitive to the glutaminase inhibitor CB-839 *in vitro* and *in vivo* (96). In contrast, Caiola et al. found that the different sensitivity to CB-839 in NSCLC was not related to genetic alteration but depended on the individual cell’s ability to generate pyruvate from alanine, to overcome the reduced availability of glutamine. They noted that the combination of the glutaminase inhibitor CB-839 with inhibition of the alanine aminotransferase using L-cycloserine, increased the tumor response to CB-839 (101).

Two phase 2 clinical trials are ongoing. The BeGin study aims to assess the response to the glutamine inhibitor telaglenastat (CB-839) in patients with solid tumors characterized by specific mutations including *KEAP1* and *STK11/LKB1* (https://clinicaltrials.gov). The KEAPSAKE trial examines the efficacy of the same glutaminase inhibitor – telaglenastat – combined with the standard-of-care pembrolizumab and chemotherapy in NSCLC patients mutated in *KEAP1* using *STK11/LKB1* status for the stratification (https://clinicaltrials.gov).

### Therapy-Induced Oxidative Stress

Reactive oxidative species (ROS) are important biological messengers that regulate different major processes including autophagy, immunity and cell differentiation (102). However, high ROS levels can cause irreversible damage, such as protein and nucleic acid oxidation, leading to cell death (103). Studies have shown it is possible to induce tumor cell death by increasing ROS intracellular levels, using different drugs.

The LKB1-AMPK pathway is directly involved in the regulation of ROS levels. In metabolic stress conditions the activation of AMPK brokered by ROS induces an PGC-1α-mediated antioxidant response (104), promotes the activation of glycolysis and the pentose phosphate pathway (105), and governs the homeostasis of NADPH (106). In addition, LKB1 regulates oxidative stress, inducing the activation of p38 and its downstream signaling targets, leading to reduction of ROS intracellular levels (39).

Given LKB1’s role in the maintenance of redox homeostasis and the ROS accumulation that characterizes LKB1-deficient tumors (76), different studies have investigated the response of this subgroup of tumors to therapy-induced oxidative stress. It emerges that cells lacking LKB1, exposed to exogenous oxidative stress, undergo mitochondrial fragmentation caused by loss of their mitochondrial membrane potential (107), accumulating macromolecular damage such as oxidation of lipids and nucleic acids, and increased cell death (76).

### Other Metabolic Agents

Several other ways to target cell metabolism in *LKB1*-deleted cancers have emerged over the years.

Kim et al., examining the possibility of selectively targeting specific subtypes of NSCLC, found that the co-mutation *KRAS/LKB1* was sufficient to make these cells dependent to the coatomer complex I (COPI) lysosome acidification. These cells rely on this process for their supply of TCA-cycle substrates by autophagy and inhibition of this mechanism, using chloroquine to inhibit lysosome acidification, induces selective death *in vivo* and *in vitro* (108).

Given the importance of lipid metabolism in maintaining of tumor growth, lipid metabolic pathways have aroused much interest as anticancer targets. Since AMPK is important even in the regulation of FA synthesis *via* the inactivation of acetyl-CoA carboxylase (ACC) (109), which catalysis the first step in this biosynthesis (110), lipid metabolism might be considered a possible target in tumors lacking LKB1. In NSCLC models chronic treatment with ND-646, an allosteric inhibitor of ACC that mimics the physiological regulation mediated by AMPK, suppresses tumor growth (111). Since loss of LKB1 causes the lack of inhibition of ACC, targeting it in LKB1-deficient tumors could be beneficial.

In NSCLC, LKB1 loss has also been linked to enhanced sensitivity to endoplasmic reticulum stress mediated by ER stress activators (ERSA) such as 2-deoxy-D-glucose, though the mechanism behind this phenotype is unclear (112).

Liu et al., using lung cancer cell lines from mouse models, identified deoxythymidylate kinase (*Dtymk*) as synthetically lethal with *LKB1* deletion. Dtymk catalysis the phosphorylation of dTMP to dTDP and its inhibition reduces the dTDP pool. *LKB1*-deleted cells were more dependent on the dTTP synthesis pathway due to the lower expression of *DTYMK*, and this might possibly explain for the greater response of these cells to the inhibition of *Dtymk*. However, unfortunately, they did not find a correlation between DTYMK levels and *LKB1* status (113).

Kim et al. analyzing tumor metabolomes from genetically engineered mouse models and gene expression profiling of human tumors co-mutated in *KRAS* and *LKB1* or *p53*, outlined a dependency of *KRAS/LKB1* mutated cells on the hexosamine biosynthesis pathway (114). In fact, the inhibition of glutamine-fructose-6-phosphate transaminase 2 (GFPT2) more potently suppresses tumor growth in cell culture, xenografts and genetically engineered mouse models of NSCLC mutated in *KRAS/LKB1* (114). The hexosamine biosynthesis pathway is a branch of glycolysis and there is increasing evidence that inhibition of this pathway may suppress tumor cell growth, enhance tumor response to conventional therapy, stimulate immune response and reduce cancer resistance, thus emerging as a possible therapeutic target (115).

Cells with the mutation of *KRAS* and deletion of *LKB1* are enriched in different enzymes implicated in the serine synthesis (78). Serine is a non-essential amino acid biosynthesized *de novo* in different cancers. The ability to activate pathways that enable cells to synthesize non-essential amino acids makes them less dependent on exogenous supplies, but may lead to selective dependency on these endogenous alterations (116).

Galan-Cobo et al. identified the 8-chloro-adenosine (8-Cl-Ado), an RNA-directed adenosine analogue that induces depletion of the endogenous ATP pool, as an active agent in LKB1-deficient NSCLC cells. These data indicate that 8-Cl-Ado activates AMPK in a LKB1-dependent manner in LKB1-proficient tumors. The lack of LKB1 results in impairment of ATP/AMP levels, making LKB1-deficient cells vulnerable to this drug (117).

## Targeting of LKB1 Downstream Pathways

In the last few years several groups have focused on LKB1 downstream pathways and their dysregulation caused by LKB1 loss, in order to find a way to target *LKB1-*mutated NSCLCs. Some strategies have been found, particularly in LKB1 downstream pathways involved in cell growth and proliferation, metastasis and DNA damage.

### Targeting LKB1 in Cell Growth and Proliferation

Through the AMPK/mTOR signaling, LKB1 is involved not only in controlling cell metabolism but also in cell growth. As explained before, in the normal wild type condition, LKB1 phosphorylates and activates AMPK that in turn activates the Tuberous Sclerosis Complex (TSC) which negatively controls mTORC1 activation (118). Gwinn et al. (119) demonstrated that AMPK can directly phosphorylate Raptor, a protein involved in the activation and activity of mTORC1. Thus, in LKB1-null cells mTORC1 is hyperactivated because of the lack of negative regulations by AMPK/TSC. mTORC1 then regulates cell growth through two proteins: the ribosomal protein s6 kinase (S6K) and the eukaryotic initiation factor 4E binding proteins (4E-BPs) which are involved in protein synthesis and translation (120, 121).

So, one of the first ways studied to treat *LKB1*-mutated NSCLC exploits the mTOR hyperactivation due to the absence of LKB1, targeting and abolishing its activity. Two different strategies to act on this complex were developed: with allosteric mTORC1 inhibitors and then with pan mTOR kinase inhibitors.

The first group of compounds comprised rapalogs, such as rapamycin, everolimus and temsirolimus which, as said before, inhibit the activity of mTORC1. They gave promising results in the treatment of cancers such as renal cell carcinoma, pancreatic tumors and HER-positive breast cancer, and were approved by the FDA for those treatments (122). However, these compounds were not so promising for lung cancer, and in general they presented problems with the development of resistance (123), for example, due to the activity of the other mTOR complex, mTORC2, which overcomes the sensitivity to these compounds. Therefore, the second group of compounds was developed - the pan mTOR inhibitors - to suppress the activity of both mTORC1 and mTORC2 (124, 125). However, the use of mTOR inhibitors creates a feedback loop that induces the activation of parallel pathways such as PI3K/AKT or MAPK/ERK1/2 (126, 127).

Different groups reported the efficacy of combinations of drugs inhibiting different proteins of these pathways. Shukuya et al. (128) demonstrated a synergistic effect of mTOR and PI3K concomitant inhibition in *LKB1* mutant NSCLC *in vitro* and *in vivo*. Carretero et al. (129) found that the concomitant inhibition of mTOR/PI3K, MEK1/2 and SRC, a non-receptor tyrosine kinase involved in cell mobility which is activated in LKB1-null cancers, can induce tumor regression and block metastasis *in vivo* (129).

In light of these findings and the modest efficacy, other strategies were examined to target *LKB1*-mutated NSCLCs, again analyzing LKB1/AMPK downstream pathways. Important results were the discoveries of MEK inhibitor efficacy on *LKB1*-mutated lung cancer. MEK is a mitogen-activated protein kinase (MAPK) belonging to the KRAS/RAF/MEK/ERK pathway. Kaufman et al. (130) identified MEK inhibitors as potential drugs for the treatment of *LKB1*-mutated lung tumors after an analysis that considered an association between the transcriptional profile of this mutated tumor and drug candidates. Mahoney et al. (131) reported the sensitivity of *LKB1/KRAS* mutated NSCLCs to the MEK inhibitor single treatment. This treatment reduces tumor proliferation probably due to the decrease in the activity of the mTOR downstream protein, p70S6K. In 2018 Wang at al (132). demonstrated the sensitivity of *LKB1/KRAS* mutated lung cancer to the combination of trametinib, a MEK inhibitor, and radiotherapy. This sensitivity was closely related to the LKB1 loss and in fact *KRAS* mutated but *LKB1* wildtype tumors are resistant to the combination. The mechanism by which *LKB1/KRAS* mutated NSCLCs are sensitive to the combination is likely to involve the ability of these cells to go to senescence, preventing the activation of autophagy. Normally when stress requires this action LKB1, through AMPK activation, can drive cells into autophagy. However, when LKB1 is lost, AMPK is not activated and cells are no longer able to induce autophagy, thus going to senescence. This is a strategy already studied for the treatment of cancer and has given promising results, especially in LKB1-null NSCLCs (76, 133). Wang et al. also showed that trametinib potentiates chemotherapy *in vivo* in *KRAS/LKB1*-mutated models.

In line with these findings, our group demonstrated another way to specifically target *LKB1*-mutated NSCLCs (134). In these tumors the inhibition of ERK, a MAPK KRAS downstream protein, reduces their growth both *in vitro* and *in vivo*, but is not effective in *LKB1* wildtype NSCLC models. Even in this case, the mechanism of this treatment passes through the mTOR pathway, particularly with the downregulation of S6K activity. However, as reported above, the tight cross-talk between LKB1, MAPK and PI3K/mTOR pathways permits a bypass of the inhibition of one protein by exploiting parallel pathways (54). We demonstrated how mutations in the PI3K pathway create resistance to ERK inhibitors, but *ad hoc* combination with a PI3K inhibitor restored the sensitivity to this class of drugs, giving them a good chance for clinical use where they are already being tested (135, 136). Finally, our data suggest that this treatment might also effective for *KRAS/LKB1* co-mutated NSCLC.

**Figure 2:**
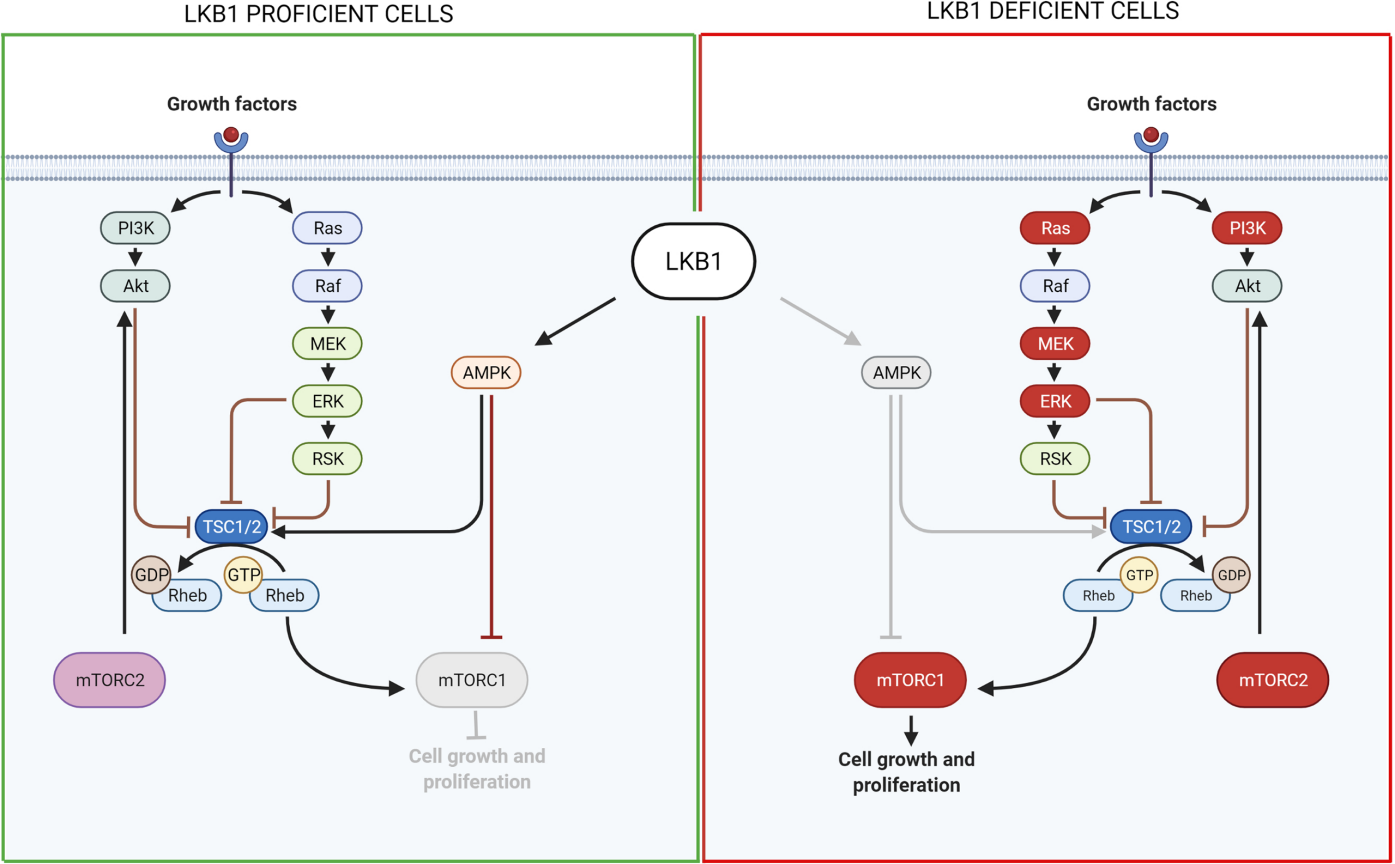
Scheme of LKB1 downstream pathways involved in cell growth and proliferation and possible targets for pharmacological inhibition of *LKB1*-mutated tumors. On the left, it is shown the pathway in LKB1-proficient cells, while on the right in LKB1-deficient cells. Grey lines and grey circle indicate the inactivation of that way. The red circle proteins on the right show the possible targets for a pharmacological inhibition of *LKB1*-mutated NSCLC growth and proliferation. Created with Biorender.com.

To conclude, all these studies indicate that the LKB1/AMPK downstream pathways passing through mTOR and controlling cell growth is an attractive candidate for targeted therapies for *LKB1-*mutated NSCLC (**Figure 2**). Moreover, many of these studies used effective drugs already in clinical experimentation, accelerating the real possibility of a specific targeted therapy for these patients. Even with the PI3K inhibitors, that cause some important toxicities, limiting their use in cancer treatment (137), the above studies demonstrated their validity in combination treatments where they should be less toxic since the doses needed in these combinations are well below the standard ones.

About 30% of KRAS-mutated NSCLC also have *LKB1* mutations and this co-presence really worsens the prognosis for patients. Many efforts have been made to find specific targeted therapies for KRAS-mutated NSCLC, highlighting the potential of Adagrasib and Sotorasib (138). Further analyses of these drugs in KRYSTAL-1 and CodeBreaK 100 trials pointed to interesting opportunities for *LKB1* co-mutated patients. Adagrasib single treatment gave a higher ORR in *KRAS/LKB1* co-mutated NSCLC, of 64%, compared to the single *KRAS* mutated ones, which reach 33% (138, 139). For Sotorasib monotherapy too in KRAS/LKB1-mutated NSCLC patients the response rate was better than in the LKB1 wildtype counterpart (140). Researchers are now verifying the efficacy of KRAS inhibitor-based drug combinations, for example, with a MEK, a mTOR inhibitor and, not least important, with a PD-1 inhibitor (138). These results offer a glimmer of hope for *LKB1*-mutated NSCLC patients, even if we have still to find out why *LKB1* mutation confers more susceptibility to the inhibitors.

Studying the others AMPK-related proteins downstream LKB1, it was discovered that the pathway LKB1-SIK, plays an important role in the regulation of tumor growth (141, 142). Even more, it seems that LKB1 controls this function by directly modulating SIKs proteins rather than through AMPK. SIKs, comprising SIK1-SIK3, are serine threonine kinases with tumor suppressive functions. It was seen, indeed, that the inhibition of SIKs functions in LKB1-proficient tumor cells leads to a cellular phenotype equal to those of LKB1-null cells, promoting tumor growth (141, 142). LKB1 controls cellular growth by phosphorylating and activating SIKs, which in turn act on cAMP response element–binding protein (CREB)–regulated transcription coactivator (CRTC) family. In particular, SIKs phosphorylate CRTCs and sequester them in the cytoplasm, where they cannot activate CREB transcription factor and the transcription of genes involved in cell proliferation (143). In LKB1-null cells, SIKs proteins are not activated, so there is an accumulation of dephosphorylated CRTCs that, moving to the nucleus, activate CREB-dependent genes such as *LINC00473*, *INSL4*, *ID1*, *NR4A1-3*, and *PTGS2* (143, 144). Thus, the loss of LKB1-SIK-CREB normal modulation induces tumor cell growth. In this context, Zhou et al. (144) showed that the activation of CRTCs is the key step in the promotion of LKB1-null tumor growth, thus the inhibition of those proteins could be a strategy to block tumor growth. Indeed, they designed a pan-CRTC inhibitor able to interfere with CRTCs binding to CREB and its activation. Finally, they demonstrated *in vitro* and *in vivo* that the use of this inhibitor significantly reduces *LKB1*-mutated NSCLC growth, without affecting *LKB1*-wildtype NSCLC models and normal lung cells. Focusing on the CREB-dependent genes, Chen et al. (145) discovered the lncRNA *LINC00473* as the most highly induced gene in LKB1-null NSCLC samples. This is essential to sustain NSCLC cell growth and survival and its high expression is due to the lack of SIKs action on CRTC-CREB. Therefore, the authors suggested it as a putative target for *LKB1*-mutated NSCLCs. Yang et al. (146) showed that *INSL4* is another gene regulated by CREB aberrant activation. It activates PI3K-AKT and MAPK pathways in an autocrine way and enhances tumor cells growth and survival. Even in this case, the authors suggest how the modulation of *INSL4* expression through an antibody or a receptor inhibitor could be valid therapeutic options. However, at present there are not specific compounds able to perform this activity.

### Targeting LKB1 to Prevent Metastasis

LKB1 plays an important role also in metastasis (8). For example, the co-mutation in *LKB1* and *KRAS* in NSCLC gives a worse phenotype, with an increased risk of metastasis (8). LKB1’s involvement in metastasis is probably related to its function in different processes such as cell polarization and motility, cell detachment, adhesion and anoikis (33). All these events are regulated by LKB1-AMPK-related protein pathways such as the LKB1-NUAK pathways involved in cell adhesion (147), the LKB1-MARKs (MAP/microtubule affinity-regulating kinases) pathway controlling cell polarization and microtubule organization (23, 148), the LKB1-SRC/FAK (focal adhesion kinase) proteins involved in cell detachment and adhesion (33, 149) and the LKB1-SIK pathway regulating anoikis and metastasis progression (141, 143, 150). All these pathways are being actively investigated. Some possible therapeutic approaches have already been suggested exploiting the LKB1-SRC/FAK and LKB-SIKs pathways.

FAK is a highly phosphorylated protein with a central role in cell adhesion, shape and migration. As a central protein it interacts with integrins and growth factor receptors, and complexes with other players to regulate, for example, the assembly and disassembly of focal contacts or the microtubule structure, all events that promote cell mobility (33, 149). Among the FAK partners, the formation of the SRC-FAK complex is important to ensure its maximal catalytic activation (149). Interestingly, an association has been reported between LKB1 and the SRC-FAK complex. The loss of the LKB1 tumor suppressor induces hyper activation of the complex, promoting the metastatic profile of LKB1-null cancers (129, 151).

**Figure 3:**
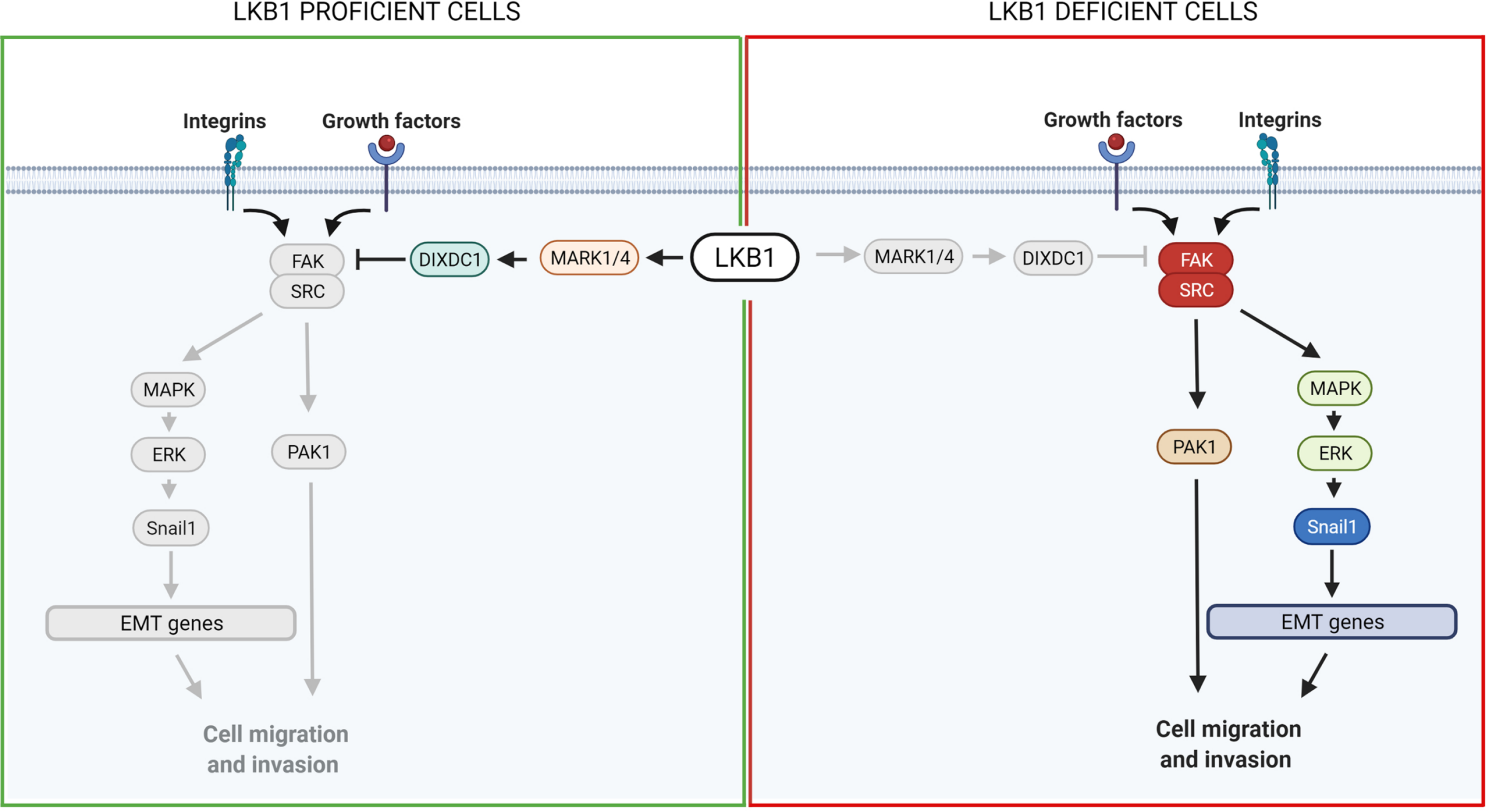
Scheme of LKB1 downstream pathways involved in metastasis and possible target for pharmacological prevention of metastases. On the left, it is shown the pathway in LKB1-proficient cells, while on the right in LKB1-deficient cells. Grey lines and grey circles indicate the inactivation of that way. The red circle proteins on the right show the possible targets for a pharmacological inhibition of *LKB1*-mutated NSCLC metastasis. Created with Biorender.com.

Different groups have studied the SRC-FAK downstream proteins. Goodwin et al. (152) discovered a link between LKB1, MARK1/4 and the transcription factor Snail1 which promotes the expression of genes involved in epithelial and mesenchymal transition (EMT), favoring metastasis. Normally, LKB1 phosphorylates and activates MARK1/4 which in turn activates DIXDC1, a scaffold protein involved in focal adhesion maturation. Inactivation of the LKB1-MARK1/4-DIXDC1 axis leads to activation of FAK which, through the MAPK/ERK1/2 pathway, upregulates Snail1 (152). Another group described a link between LKB1, SRC/FAK and PAK1: LKB1 negatively regulates PAK1 inhibiting cell mobility while SRC-FAK acts in the opposite way, activating PAK1 and hence migration. In LKB1-null cells, therefore, the SRC-FAK hyperactivated axis promotes cell mobility activating PAK1 (153) (**Figure 3**).

Therefore, after the discovery of the crosstalk and relation between LKB1 and SRC/FAK and their important role in cell mobility, this pathway became an interesting potential target for *LKB1*-mutated NSCLC therapies. At least two groups have shown a possible efficacious way to develop a targeted therapy. Carretero et al. demonstrated a susceptibility of LKB1-null NSCLC cell lines to RNAi-mediated signaling of SRC and FAK and the ability of the SRC inhibitor Dasatinib or the FAK inhibitor PF 573228 to reduce cell adhesion and migration (129, 154). As reported above, the authors also showed how Dasatinib in combination with a PI3K/mTOR inhibitor and a MEK inhibitor restored the sensitivity of *KRAS/LKB1*-mutated tumors to this combination, otherwise active only in *KRAS* mutated *LKB1* wildtype tumors (129).

Gilbert-Ross et al. (155) also illustrated the potential of FAK inhibitors in clinical use for *KRAS/LKB1* co-mutated NSCLCs. They performed a series of analyses on a lentiviral-Cre-induced *KRAS G12D LKB1*-mutated genetically engineered mouse model (GEMM) showing high levels of FAK autophosphorylation at site 397 (pYFAK397), a signal of high activity of this protein, and that this upregulation reflects in the collective invasion of the surrounding collagen. In addition, they show that treatment with two FAK inhibitors in LKB1-null models suppressed FAK activity and the tumor’s tendency to collective invasion. In addition, treating these GEMMs at an early stage with a FAK inhibitor, GSK6098, there was a significant reduction in tumor burden and in invasive behavior.

Overall, targeting SRC/FAK might be a promising way to develop effective therapies for *LKB1*-mutated NSCLC, although further studies are needed to assess their real efficacy in human clinical trials and in different subtypes of *LKB1-*mutated or co-mutated NSCLC (155). Several phase I clinical trials with a FAK inhibitor in solid cancers are ongoing (https://clinicaltrials.gov), so they could have a bright continuation.

Regarding LKB1-SIKs pathway, it was discovered that it plays an important role in driving metastasis. The dysregulation of LKB1-SIK-CREB axis induces the expression of the *inhibitor of DNA binding 1* (*ID1*) (156), an oncogene already studied and demonstrated to be essential for lung colonization by breast cancer (157). In NSCLC, this gene controls cell-cell and cell-ECM interaction, cytoskeleton and other aspects involved in anchorage-independent growth and colonization promoting in this way metastasis. Therefore, inhibitors of ID proteins, already developed, could be a viable therapeutic option. Moreover, in normal condition, SIKs regulate transcription factors that lead to E-cadherin expression and thus epithelial to mesenchymal transition (EMT) block. In LKB1-null cells this inhibition is lost and contributed to NSCLC invasion and migration (143). Again, dysregulation of LKB1-SIK-p53 axis inhibits the anoikis (apoptosis induced by cell detachment) and increases metastasis (150). Finally, SIKs act also on inhibition of class IIa histone deacetylases (HDACs) that interact with chromatin. Abnormalities in LKB1-SIK-HDAC pathway induce chromatin changes and epigenetic regulations which enhance tumor cells metastatic ability (158).

### Targeting LKB1 in DNA Damage

**Figure 4:**
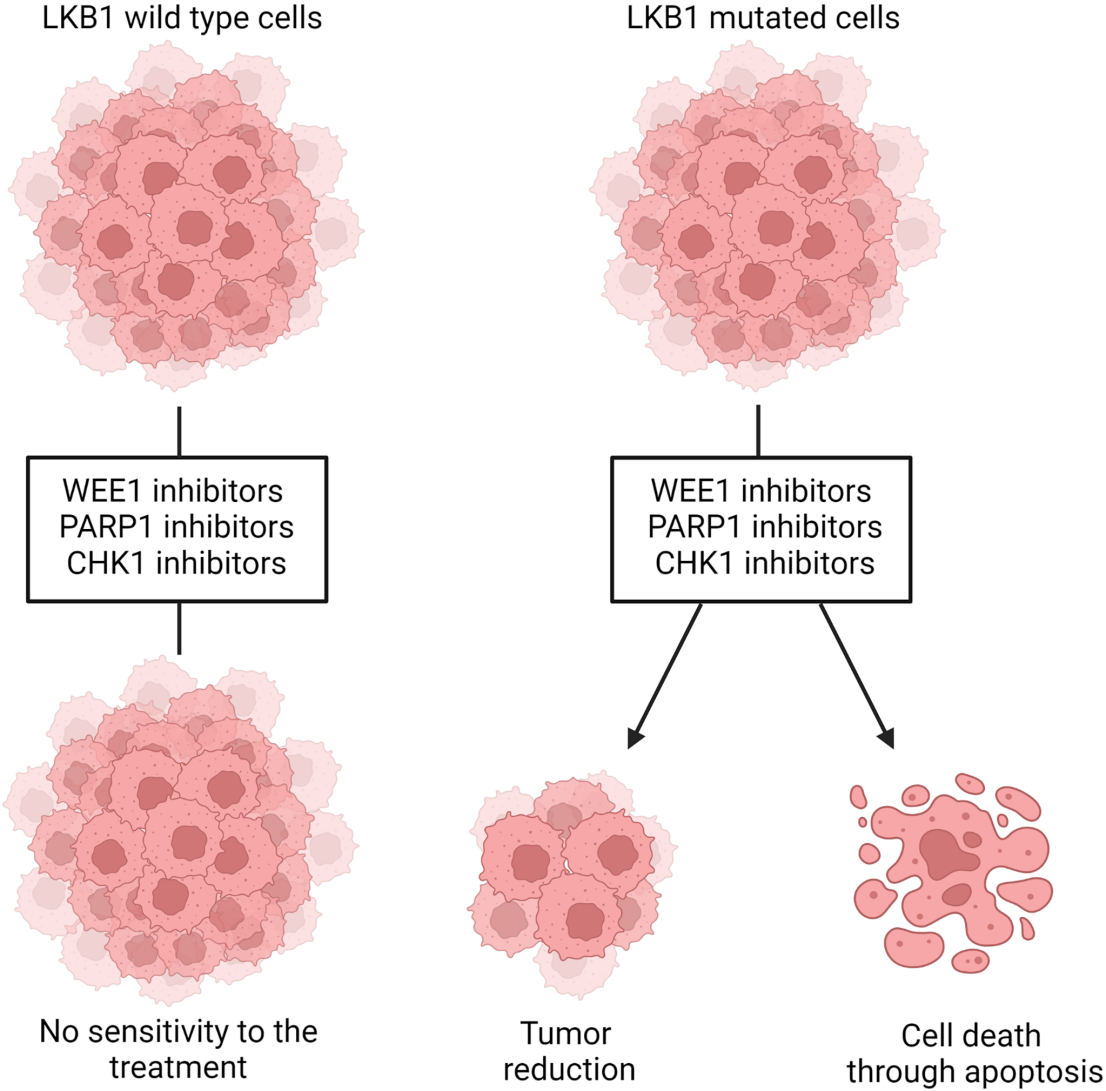
Representation of WEE1, PARP1 and CHK1 inhibition in *LKB1* wildtype and mutated cells. *LKB1* wildtype cells are not sensitive to the treatment with those inhibitors while, in *LKB1*-mutated cells, they leads to decreased cell viability and tumor size and/or increased DNA damages that, in turn, induce cells death through apoptosis. Created with Biorender.com.

LKB1 is also involved in the maintenance of DNA integrity and prevention of DNA damage, when it is localized in the nucleus. The precise mechanism through which LKB1 participates in this fundamental process is not completely clear. A connection between ataxia telangiectasia-mutated kinase (AT) which together with ATR and DNA-PK act as a DNA damage checkpoint, that controls the progression of the cell cycle [or blocks it if DNA damage is detected (159, 160)], and LKB1 has been reported (41, 161). In the presence of DNA damage ATM is activated and phosphorylates LKB1 at the T363 residue (161, 162). This mechanism seems to underlie the susceptibility of *LKB1/KRAS* co-mutated NSCLC cells to a WEE1 inhibitor, AZD1775, reported by Richer et al. (163). They noticed that, despite the equal downregulation of phosphorylated CDC2, a target of WEE1 kinase, treatment with the WEE1 inhibitor significantly reduced the cell viability and increased the DNA damage, just in LKB1-null NSCLC cell lines, in an AMPK-independent way (**Figure 4**). They also demonstrated that in these LKB1-null cell lines only, the combination of DNA-damaging agents such as cisplatin to AZD1775 increased cell mortality through apoptosis. This combination *in vivo* prolonged the survival of *KRAS/LKB1-*mutated NSCLC mice. The WEE1 inhibitor has already been tested in clinical trials, is well tolerated and gives positive results in patients with cancer defective in DNA damage repair or G1 checkpoint aberration (164, 165). Further studies with this inhibitor for LKB1-null NSCLC patients are now warranted for the development of effective targeted therapy.

Beyond the evidence of ATM-LKB1 crosstalk, LKB1 is also involved in homologous recombination (HR), directly interacting with BRCA1, thus the lack of LKB1 could impair this way to repair DNA damage (40). Therefore, exploiting this in LKB1-null tumors, blocking mechanism of DNA repair (i.e. base excision repair, BER) is likely to increase the DNA instability, hence cell death. PARP-1 is the main enzyme involved in BER, and the use of PARP-1 inhibitors in LKB1-null NSCLC cell lines significantly reduced their growth (40) (**Figure 4**). In support of this preclinical study, a clinical trial is now active using a PARP-1 inhibitor, the LUNG-MAP treatment trial. This trial evaluates the response of patients with stage IV or recurrent non-squamous NSCLC with *LKB1* gene mutation to the combination treatment based on a PARP-1 inhibitor, Talazoparib, and Avelumab, a PD-L1 monoclonal antibody (https://clinicaltrials.gov).

There may be other ways in which LKB1 is involved in the DNA damage response. Xiaoduo Xie et al. demonstrated that the mTOR downstream protein S6K, phosphorylating the E3 ubiquitin ligase RNF168 which is important for polyubiquitination of histone at DNA damage sites, inhibits its activity, impairing the response to DNA damage (166). There is also an association between LKB1 status and RNF168 abundance. To the best of our knowledge, however, no studies have exploited this pathway to seek a *LKB1*-mutated NSCLC specific therapy.

Liu et al. (113) did shRNA screening on LKB1-null NSCLC cells in order to detect the genes acting in synthetic lethality with LKB1. They found Chek1 and Dtymk genes, both involved in the regulation of DNA damage. DTYMK, with its function in converting dTMP in dTDP, is involved in DNA synthesis, hence cell growth. When DTYMK is absent, there is not only a deficiency of dTTP but also an accumulation of dUTP which is incorporated in DNA, causing damages. The authors found that LKB1-null NSCLC cell lines were sensitive to the knockout of DTYMK, indicating this protein as a possible target (113). However, this research has not produced any further relevant data. On the other hand, the same group reported encouraging data about the inhibition of CHK1 in LKB1-null NSCLC. The CHK1 inhibitor reduced the size of *LKB1-*mutated NSCLC both *in vitro* and *in vivo* (**Figure 4**). This vulnerability of LKB1-null NSCLC is interesting in view of the established clinical use of both gemcitabine and CHK1 inhibitors in clinic. In fact, the CHK1 inhibitor significantly synergizes with gemcitabine (167).

## LKB1 and Immune System

The introduction of immune checkpoint inhibitors (ICIs), as monotherapy or in combination with chemotherapy, has had a great clinical impact in the treatment of NSCLCs, resulting in important improvement in overall survival. However, treatment resistance or toxicity means that many patients do not benefit from ICIs (53).

Many retrospective clinical studies investigated *LKB1* mutations as a negative determinant of immunotherapy response (50, 168–170). Indeed, patients with non-squamous *LKB1*-mutated NSCLC tumors do not respond to ICI or they general have dramatically lower survival and progression-free survival (50, 171, 172). This is true for patients given with ICI both in first-line and second-line treatments, and considering both monotherapy and a combination of two ICIs (168, 173, 174). However, studies in which the LKB1 mutant tumors’ response to ICI was compared to that of chemotherapy, showed a similarly lower response to both treatments (43, 175). Therefore, *LKB1* mutations should probably be considered a prognostic factor, independently from the treatment type, rather than a predictive biomarker of ICIs resistance.

In the last few years much efforts have been made to elucidate the biological alterations leading to immunotherapy resistance in *LKB1*-mutated tumors. LKB1 is involved in the regulation of T regulatory and T effector cell activity, in the maintenance of survival and proliferation of peripheral T cells and in promoting thymocyte development (176–178). Furthermore, there is growing evidence of the role of LKB1 in regulation of the immune microenvironment. *LKB1* inactivation in NSCLC is associated with a reduction of CD8+ T lymphocytes and a higher density of the immune-suppressive cell population, defining an “inert” or cold tumor immune microenvironment, that promotes resistance to PD-1/PD-L1 inhibitors (43, 50).

A significant role of cytokines and chemokines in *LKB1*-deleted tumors has been described by Koyama and colleagues (46). Investigating the effects of LKB1 on the tumor microenvironment, they found that its inactivation promoted greater expression of neutrophil-recruiting chemokines such as CXCL7 and CXCL5 and pro-inflammatory cytokines such as IL-6, which contributes to neutrophil accumulation in NSCLC models. In addition, there was lower expression of lymphocytes and dendritic cells recruiting chemokines such as CCL5 and CXCL12, resulting in under-representation of these cell types in the absence of LKB1. Blocking IL-6 inhibited tumor proliferation and increased T cell function in a *LKB1*-mutated mouse model, suggesting that aberrant cytokine signaling could be a promising immunotherapeutic strategy in selected patients (46). In line with these findings, Li et al. found that loss of LKB1 induced a greater production of the ELR+ CXC chemokines in lung cancer models. The high level of these chemokines correlates with enrichment of granulocytic myeloid-derived suppressor cells (G-MDSC), both in the tumor microenvironment and systemically. Depletion of G-MDSCs obtained using anti-Gr-1 antibody or all-trans retinoic acid (ATRA) had the result of reversing the immune suppression and sensitized LKB1-deficient tumors to immunotherapy, suggesting that ATRA therapy might offer useful strategy to overcome immune resistance in NSCLC deleted in *LKB1* (179).

**Figure 5:**
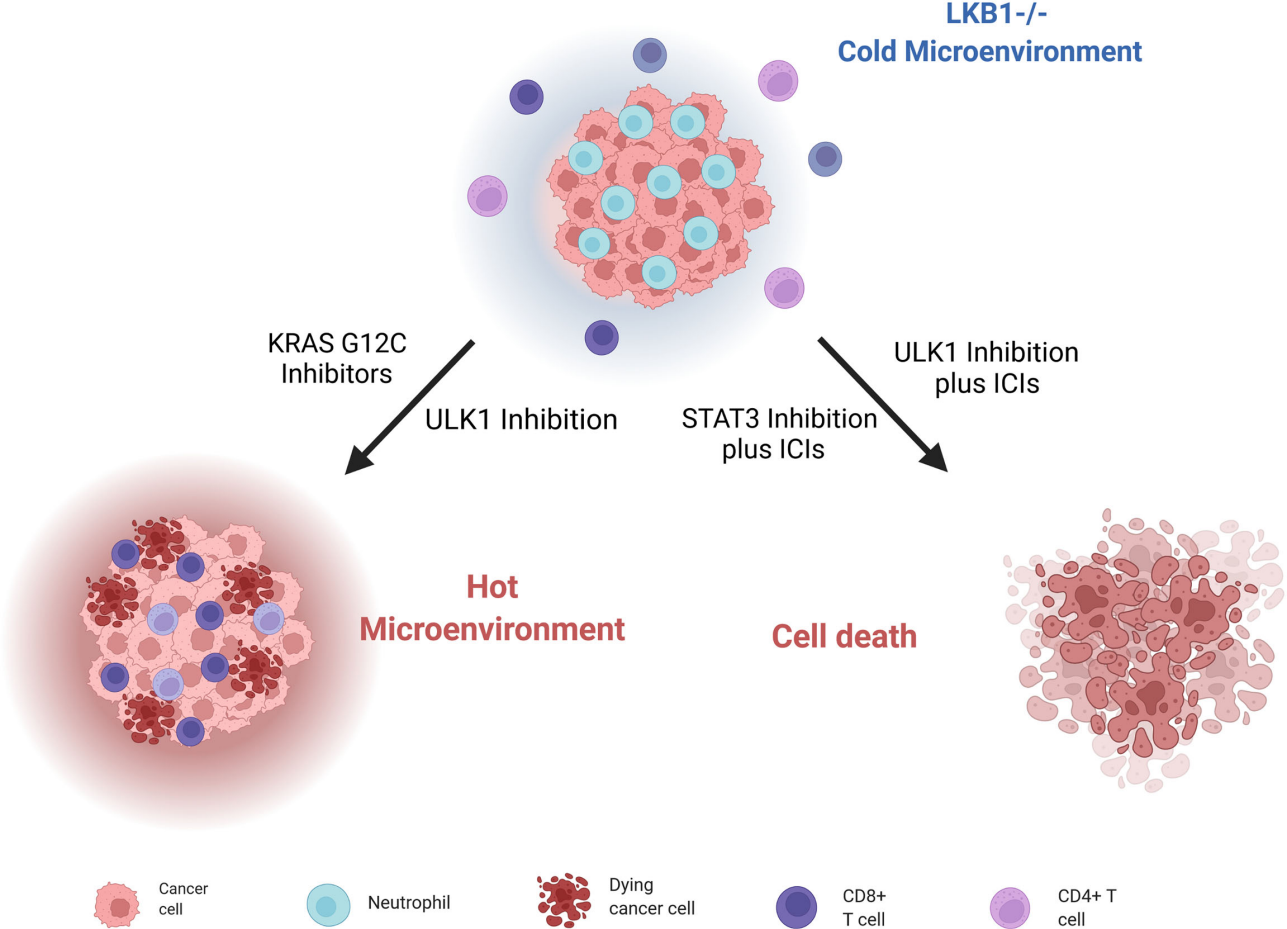
Scheme of *LKB1*-deleted cells immune microenvironment and different strategies to render them sensible to ICIs treatment and stimulate immune cells. Created with Biorender.com.

As mentioned, *LKB1*-mutated tumors have high levels of chemokines and cytokines that stimulate their respective receptors CXCR2, G-CSFR, IL6R, IL10R and so on. Signals from these receptors lead to the activation of STAT3 transcriptional factor, which give rise to an immunosuppressive phenotype. Pore et al. demonstrated that the inhibition of STAT3 with an antisense oligonucleotide (ASO) induced a series of modifications in the tumor environment (mostly by increasing the antigen-presenting cells’ activity), that leads to inhibition of tumor growth. STAT3 ASO is also effective as monotherapy in *LKB1*-mutated preclinical models but even better it has an additive effect when combined with ICI inhibitors, with the strongest activity in triple combination with PD-L1 and CTLA4 inhibitors (45) (**Figure 5**).

Therefore, even if mutations in the *LKB1* gene are generally correlated with resistance to PD-1/PD-L1 and CTLA4 inhibitors, the concomitant use of other targeted agents could reverse this resistance, making also this tumor eligible for immunotherapy.

Moreover, Deng et al. (180) discovered that patients with *KRAS* and *LKB1*-mutated (KL) NSCLC, together with KL murine cancer cell lines and genetically engineered mouse models (GEMMs) of lung cancers, have a low immunoproteasome activity and increased autophagy flux; these features compromise the processing and presentation of antigens to MHC-1, leading to evasion of the immune response by tumor cells. Reversing this altered cell environment has given positive preclinical results, suggesting it as valid strategy to treat *LKB1-*mutated NSCLCs. Indeed, blocking autophagy through inhibition of ULK1, a protein fundamental for autophagy to start (181), restores the immunoproteasome activity and antigen presentation and leads to increasing CD4+ and CD8+ T cell infiltration, which is normally low in LKB1-null NSCLCs. The combination of an ULK1 inhibitor and a PD-1 inhibitor sensitizes these tumors to ICI treatment (**Figure 5**).

Since the key role of the immune microenvironment in the onset and progression of cancer has emerged, different strategies to stimulate the immune response have been investigated. Promising results have emerged with KRAS^G12C^ inhibitors in NSCLC; in fact, targeting mutant KRAS can reverse the cancer cells’ ability to avoid immune surveillance, and can enhance an immune-attractive microenvironment (182) (**Figure 5**). In this scenario, NSCLC patients bearing the co-mutation *KRAS^G12C^*/*LKB1^del^* treated with a KRAS^G12C^ inhibitor have given a stronger response than to other subgroups (183). These results lay a basis for promising new therapeutic strategies.

## Discussion

LKB1 is an ubiquitous protein involved in several important cellular processes and its loss of function has dramatic consequences in the aggressiveness of cancers. Moreover, because almost all LKB1 mutations lead to the lack of the relative protein, it is really hard to target *LKB1*-mutated cancers, like *LKB1*-mutated NSCLC. However, as reported in this review, on the one hand, it could be possible to exploit the cellular metabolism alterations due to the loss of LKB1 to specifically treat this tumor. On the other hand, strategies used to find targetable pathways in *LKB1*-mutated cancer, such as the use of FDA-drug or Crispr-CAS9 libraries screening have shown the possibility of using specific protein inhibitors to hit this cancer. Finally, in the last few years many researchers have investigated the implications of LKB1 in immunotherapy resistance, and some preliminary studies point to ways to restore the “heat” in this ‘cold’ tumor.

Therefore, despite the difficulties in targeting a hidden target and the huge implications of its absence, viable strategies have been indicated and the subsequent deeper research means that some of them could really be a breakthrough for patients with *LKB1*-mutated NSCLC.

## Author Contributions

GN, MM, EC, PM, LR, MB and MC contributed conception and design of the review. GN, II, EP, EC and MC wrote the manuscript. All authors contributed to manuscript revision, read and approved the submitted version. This work was supported by la Fondazione Italiana per 'La Ricerca sul Cancro – AIRC’ IG 2020 ID. 243447 project -Principal Investigator: MB

## Funding

The generous contribution of the Associazione Italiana per la Ricerca sul Cancro, through grant IG-24347, is gratefully acknowledged.

## Conflict of Interest

The authors declare that the research was conducted in the absence of any commercial or financial relationships that could be construed as a potential conflict of interest.

## Publisher’s Note

All claims expressed in this article are solely those of the authors and do not necessarily represent those of their affiliated organizations, or those of the publisher, the editors and the reviewers. Any product that may be evaluated in this article, or claim that may be made by its manufacturer, is not guaranteed or endorsed by the publisher.
